# Supplementary material for: Cholesterol Dependent Activity of the Adenosine A2A Receptor Is Modulated via the Cholesterol Consensus Motif
Source: Molecules. 2022 May 31;27(11):3529. doi: 10.3390/molecules27113529 (PMC9182133; doi:10.3390/molecules27113529)
Supplement: Supplementary file 1 [file molecules-27-03529-s001.zip › molecules-1713903-supplementary.pdf]

molecules

Supplementary Material for "Cholesterol Dependent Activity of the Adenosine A_{2A} Receptor is Modulated via the Cholesterol Consensus Motif"

By Claire McGraw, Kirsten Swonger Koretz, Daniel Oseid, Edward Lyman, and Anne Skaja Robinson*

Methods

Protein expression quantification. Isolated cell membranes were utilized for $A_{2A}R$ protein quantification via western immunoassay. 10 μg of total protein per sample was loaded onto a 12% Tris-Glycine gel and electrophoresed in SDS buffer at constant 125 V for 65 minutes. Western immunoassay was performed using adenosine A_{2A} rabbit polyclonal IgG antibody (sc-13937; Santa Cruz Biotechnology, Dallas, TX) at 1:5000 dilution, and Dnk pAb to Rb IgG HRP antibody (ab16284; Abcam, Cambridge, MA) at 1:5000 dilution. Membranes were imaged with the UVP BioSpectrum imaging system.

Plasma Membrane Staining. To confirm trafficking of WT $A_{2A}R$ and CCM variants to the plasma membrane, a membrane impermeable dye (WGA Alexa Fluor 555 conjugate, Molecular Probes, Eugene, OR) was utilized. For experimentation, cells were seeded at a density of 0.1 x 10^6 cells/ confocal well into a confocal imaging chamber (Nunc Lab-Tek II chambered cover glass, Thermo Scientific). The following day cells were transfected using 250 ng of either pCEP4-A_{2A}R-CFP, pCEP4-S47A-CFP, pCEP4-K122A-CFP or pCEP4-W129A-CFP plasmid DNA and 1 μl Lipofectamine 2000 reagent per confocal well according to manufacturer's protocol. 24- 48 hours post transfection cells were incubated with 2 μg/ ml WGA 555 in 300 μl PBS for 5 minutes at 37°C, and protected from light, as previously describe [38]. Following the 5-minute incubation, the dye was carefully aspirated out, and replaced with 100 μl PBS for imaging.

Confocal Microscopy. All confocal images were acquired on an inverted Nikon A1 confocal microscope using a 20X objective.

Analysis of Receptor Expression and Trafficking. Images of cells transiently transfected with CFP tagged receptor and stained with a plasma membrane dye were used to determine changes in localization of the receptor. Images were cropped to contain only one cell per file, and only images where the plasma membrane dye remained at the membrane and did not stain intracellular organelles were used for analysis. Each file was converted into two 8-bit grayscale images, one containing an image of the CFP tagged receptor, and the other the plasma membrane dye. In order to determine if CFP was localized at the plasma membrane, the modified directed Hausdorff distance $\widetilde{H}\,(A, B)$ assigns to every point a its nearest neighbor b in point sets A and B as previously described [38].

$$\widetilde{H}\,(A, B) = average_{a\in A}\, min_{b\in B}\, \|a - b\|$$

where $\|a - b\|$ denotes the distance between points a and b. A small Hausdorff distance (close to 0) signifies localization of the CFP tagged receptor close to the plasma membrane. A larger Hausdorff distance correlates to an increase in intracellular localization of the receptor. Images were cropped and analyzed in Fiji, and the Hausdorff distance was computed with a java plug-in provided by Dr. Carola Wenk (Tulane University).

Active G protein Western blot Purified $G\alpha_s$ activity was confirmed by $G\alpha_s$ Activation Assay Kit, carried out per manufacturer's instructions (NewEast Biosciences, King of Prussia, Pennsylvania). The $G\alpha_s$ Activation Assay Kit detects G protein activity using a monoclonal antibody that specifically binds to $G\alpha_s$ only when GTP is also bound. Inactive $G\alpha_s$ cannot bind GTP, and this monoclonal antibody has a much lower affinity for $G\alpha_s$

without GTP bound (e.g., Gαs-GDP). Briefly, 500 µL of 1 µM purified Gαs was aliquoted into two microcentrifuge tubes and mixed with 5 µL of 1 M $MgCl_2$, and 5 µL of either 100X GTPγs (positive control) or 100X GDP (negative control). Samples were then incubated at 30°C for 90 minutes on a rocker. After incubation with GTPγs or GDP, 40 µL of 1X Assay/Lysis buffer was added to a total volume of 1 mL. Samples were mixed with 1 µL of anti-active Gαs monoclonal antibody and 20 µL of resuspended protein A/G agarose bead slurry, and following a centrifugation and wash step, washed beads resuspended with 20 µL of 2X reducing SDS-PAGE sample buffer and boiled for 5 minutes. Here, 15 µL of the supernatant was transferred to a fresh microcentrifuge tube and mixed with 4X Laemmli buffer and electrophoresed through a 12% polyacrylamide gel (BioRad) at constant voltage for 60 min at 125 V. Separated proteins were transferred to a PVDF membrane (Trans-Blot Turbo Transfer System, Biorad) following manufacturer's directions. Membrane was incubated with 5% non-fat dry milk in TBST (Blocking buffer) for 1 h with agitation to reduce non-specific binding. Anti- Gαs monoclonal antibody at 1:1000 was added to the Blocking buffer and incubated with agitation for 1 h. Following incubation with the primary antibody, the membrane was washed 3X with TBST for 5 minutes per wash before adding the secondary antibody goat pAb to Ms IgG HRP (ab97265; AbCam) at a 1:3000 dilution for 1 h. Following washing, chemiluminescence detection was employed on a ChemiDoc MP imaging system (BioRad).

Table S1. Site-directed variants of $A_{2A}R$ at the CCM.

Variant Name	WT Codon	Variant Codon
S47A	TCA	GCA
K122A	AAG	GCG
W129A	TGG	GCG

Table S2. List of primers used for site-directed mutagenesis of CCM variants

Oligonucleotides		Sequence (5' –> 3')
S47A	Forward	GCCGCCGCCAGTGCCACCACAAAGTAG
	Reverse	CTACTTTGTGGTGGCACTGGCGGCGGC
K122A	Forward	GCAATGATGCCCGCAGCCCTCGTGCCGGTCA
	Reverse	TGACCGGCACGAGGGCTGCGGGCATCATTGC
W129A	Forward	GGCAAACGACAGCACCGCGCAGATGGCAATGATG
	Reverse	CATCATTGCCATCTGCGCGGTGCTGTCGTTTGCC

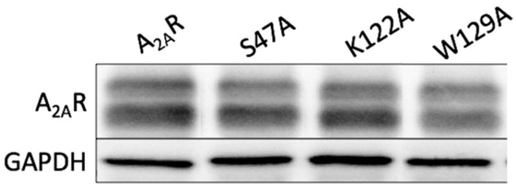

Figure S1. Protein expression quantification of CCM variants. Western blot of membrane preparations from HEK293 cells expressing $A_{2A}R$ and each CCM variant for the expression of $A_{2A}R$ and GAPDH. Doublet for the band for $A_{2A}R$ has been shown previously in our laboratory to result from glycosylation of the receptor (McCusker, PhD thesis, 2008)

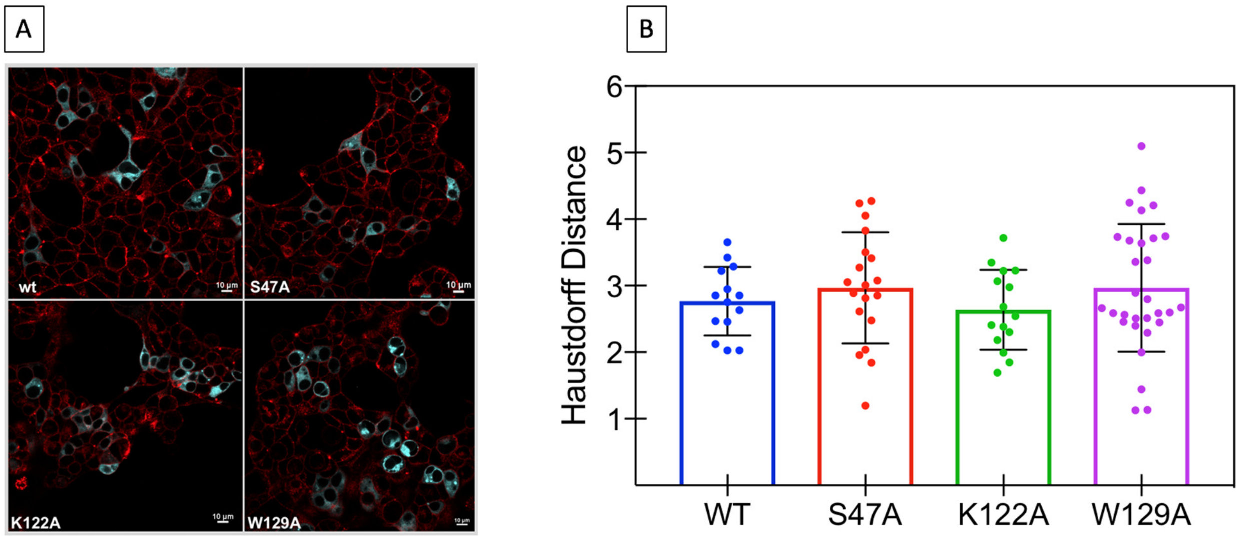

Figure S2. Protein localization of $A_{2A}R$ and CCM variants. (**A**) Representative confocal microscopy images to show localization of $A_{2A}R$ and CCM variants tagged with CFP, and stained using WGA 555 (a plasma membrane dye, shown in red). Scale bar shows 10 µM. (**B**) Calculated Hausdorff distance to determine receptor localization. Values calculated for $n \geq 14$ cells plotted individually in color. Bars represents mean ± S.D.

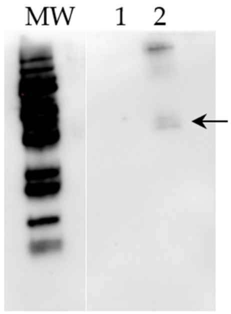

Figure S3. G protein activity Western blot. Western blot revealed activity of purified $G\alpha_s$ bound to GDP (lane 1; negative control) or GTPγs (lane 2). Here, purified $G\alpha_s$ associates with GDP and GTPγs, and the $G\alpha_s$-GTPγs complex (arrow) is visualized using the $G\alpha_s$ activity kit. The ladder is Precision Plus Western C.

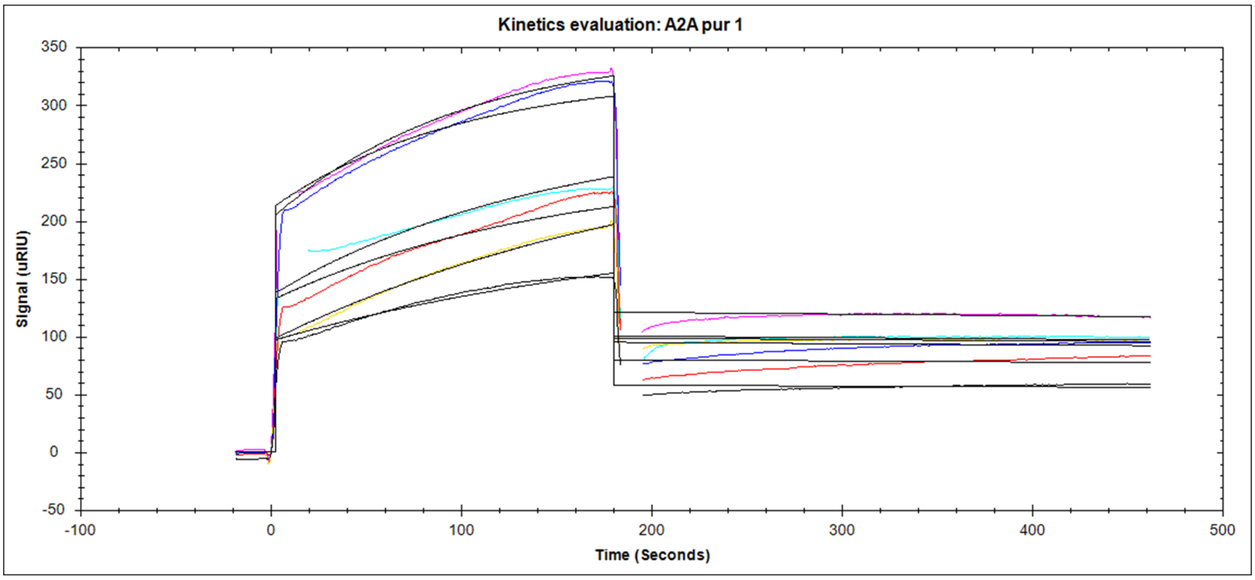

Figure S4. Representative SPR fitted data of one purification of $A_{2A}R$ to associating to $G\alpha_s$. Purified $G\alpha_s$ was attached to Ni-NTA chip and 296, 445, and 667 nM of purified $A_{2A}R$ was flowed across G protein. Each purification of $A_{2A}R$ or variant associating to G protein was analyzed using TraceDrawer and curves fit to a One-to-One binding model.

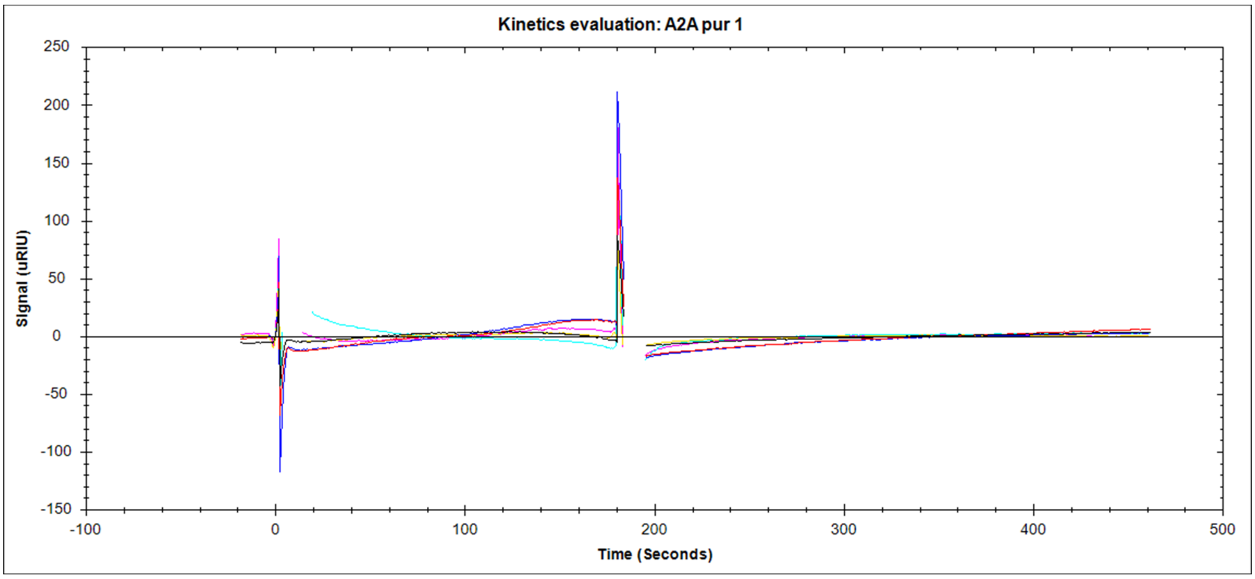

Figure S5. Residuals of SPR fits. The difference between the raw data and the model fit are shown as residuals. This is a representative example, based on Figure S4 data, but reflects typical values. Note that the observed peaks result from a bulk shift due to sample injection.
